# Internet gaming disorder behaviours: a preliminary exploration of individualism and collectivism profiles

**DOI:** 10.1186/s12888-021-03245-8

**Published:** 2021-05-20

**Authors:** Vasileios Stavropoulos, Tyler Michael John Frost, Taylor Brown, Peter Gill, Trent Anthony Footitt, Lee Kannis-Dymand

**Affiliations:** 1 Institute for Health and Sprot, Victoria University, Melbourne, Australia; 2 University of the Sunshine Coast, Queensland, Australia

**Keywords:** Internet gaming disorder, Individualism, Collectivism, Culture, Latent class analysis, Internet Gaming Disorder Behaviours: A Preliminary Exploration of Individualism and Collectivism Profiles.

## Abstract

**Background:**

Internet Gaming Disorder (IGD) behaviours involve excessive use of Internet games to the extent that ones everyday life is compromised. It has been suggested that IGD symptoms are dependent on whether one is more individualistic or collectivistic. However, the range of potential individualism-collectivismeffects on IGD presentations remains largely unknown. The current study aims to further understanding of the role of individualistic and collectivistic tendencies in IGD, allowing us to develop more gamer centredIGD prevention and intervention strategies.

**Methods:**

One thousand and thirty-twocommunity participants withinternet gaming experience were measured online for IGD symptoms severity using the Internet Gaming Disorder Scale Short Form (IGDS9-SF) andindividualism-collectivism behavioursvia the Individualism & Collectivism Scale (ICS). Latent Class Analysis (LCA) and T-Tests were performed in relation to their responses.

**Results:**

Upon inspection of the LCA output, two profiles of internet gamers were identified. These includedthe Collectivism Aversivegamers (CA; 11%) and the Collectivism Neutral gamers (CN; 89%). The CA gamers displayed significantly higher IGD behaviours overall, and, were higher inpreoccupation, withdrawal symptoms, tolerance, relapse, deception, escapism/mood modification, and functional impairment compared to CN gamers. There were no differences between CA and CN gamers in loss of interest and conflicts with others.

**Conclusions:**

The findings suggest that ones individualism-collectivism orientation can influence the presentation of IGD. Specifically, those who are less collectivistic or are less influenced by social groups willdisplay greater IGD symptoms and present a profile that requires a different intervention from gamers who are more collectivistic. Researchers and clinicians should emphasize the value of belonging in a collective and experiencing equality with others in relation to mental health and gaming patterns.

**Supplementary Information:**

The online version contains supplementary material available at 10.1186/s12888-021-03245-8.

## Background

Digital gaming refers broadly to any video game, electronic and/or interactive, which requires a visual interface, such asa personal computer monitor, television and/or a cellular screen [1]. Since its inception in the 1950s and its first mainstream boom in the 19801990s, digital gaming has grown rapidly [2]. Online gaming has also transcended the notion of personalised and isolated digital gameplay for leisure [3,5]. Online gaming can be similar to many other digital games, where an individual participates in a visual-audio simulated game-platform, with the additional requirement of internet connection to access this world [6]. Approximately 2.5 billion people (a third of the worlds population) are currently gaming in some form or frequency [2], while 56% of gamers partake at least once a week with an average playing time of 7h [2].

These developments have prompted considerations about how ones self and their surroundings may be positively and/or negatively affected by internet gaming [7,18]. Indeed, gaming participation has been shown tolikely enhance ones sense of belonging, purpose and achievement, dexterity skills development, and their socialization [19]. However, some gamers have been supported to game onlineto escape from life stressors [7]. Over-reliance on such behaviours may compromise their well-being in the longer term [3,5]. This may occur through withdrawal from other life events (e.g. work, education, relationships, family, etc.) and a gamers loss of passage of time whilst playing [3, 4]. Although such behaviours appear to be normally (i.e. only a minority present with extreme behaviours) and transiently (i.e. not permanently) distributed along the general population of internet gamers; when they escalate and persist, they underpin onesinability to control their use [20]. The latter has been linked with a range of negative behaviours [7]. These include, whilst not limited to, increased experiences of depression, anxiety and inattention, reduced social relationships, employment and educational performance, as well as comorbiduse of alcohol and/or substances [21].

### Disordered gaming diagnosis

The American Psychiatric Association (APA [22];) and the World Health Organization (WHO [23];) have begun to group these negative consequences together as related to a clinical disorder, within the spectrum of addictions. An addiction refers to symptoms associated with excessive and/or problematic use of a substance or engagement in an activity, where one loses control over their behaviour, despite the harmful consequences this entails (e.g. substance abuse disorder; gambling disorder) [22]. Some scholars have suggested that addictions (independent of their specific content) share six distinct components involving, salience (i.e. persistent reoccurrence), tolerance (i.e. higher dosages of the behaviour are prospectively required), mood modification (i.e. one does that to change how they feel), relapse (i.e. one is unable to control/abstain from the behaviour), withdrawal (i.e. reducing or eliminating the behaviour invites physiological and/or psychological discomfort), conflicts (i. e. ones surrounding is opposing the behaviour and tensions arise), and functional impairment (i.e. ones daily activities, employment and/or education is negatively impacted) [24, 25]. Specifically, the APA, prompted by reported clinical and research developments, identified excessive gaming behaviours, as a conditional mental disorder in the appendix of the Diagnostic and Statistical Manual of Mental Disorders 5th edition (DSM-5 [22];), under the term of Internet Gaming Disorder (IGD). The conditional IGDdefinition consists of 9 criteria/behaviours, whereby if an individual experiences at least five symptoms, potential diagnosis could be inferred. The DSM-5 outlines the list of criteria that is imperative for an individual to be diagnosed with IGD, including: (i) gaming preoccupation, (ii) withdrawal symptoms, (iii) gaming progressively increasing over time, (iv) escapism motivations, (v) unsuccessful gaming control, (vi) continuing gaming despite risk awareness, (vii) loss of interest in other life events, (viii) deception about game time and (ix) jeopardising significant relationships because of gaming. Some years later, the WHO [23], aligning to some extent with the APA [22], officially launched Gaming Disorder (GD) as a diagnostic classification into the 11th revision of International Classification of Diseases (ICD-11) as a formal mental health disorder. In the ICD-11, GD is broadly defined as a persistent gaming pattern and/or recurrent gaming behaviours that undermine ones everyday life [23]. Moreover, GD is explained in the ICD-11 as being manifested by: (i) impaired gaming control (for example: onset, frequency, intensity and duration), (ii) gaming priority over life events (for example: work, education or personal commitments) and (iii) gaming escalates despite negative consequences [23]. At this point it is noted, that the IGD term and criteria [22] will be followed in the present study. This choice is largely driven by: a) the existence of well-established instruments to assess IGD, which have demonstrated comparability of ratings across different cultural populations [26, 27] and; b) the need to align our findings with a significant body of international research that has employed the IGD terms and measurement [10, 28, 29].

### Criticism of the disordered gaming diagnosis

Despite the progress made, several criticisms persist in relation to the IGD diagnosis [30,32]. Firstly, the risk of pathologizing normal entertainment behaviours such as internet gaming has been illustrated [30]. Indeed, gaming onlinein a regulated manner for recreation and entertainment purposes has been suggested not cause any harm or impairment to ones life, and this presents to have been the case for the majority of those involved [33]. Secondly, it is argued that excessive/disordered gaming may not justify as an independent psychopathological diagnosis, as in most of the cases it is either comorbid and/or presents to be a secondary symptom related to other primary mental healthdisorders (i.e. anxiety, depression, addictions) [7, 11, 34,37]. Thirdly, it is argued that the definitions provided may pathologize the medium (i.e.internet gaming), whilst what is more important is the user himself/herself in terms of the risk of developing an excessive gaming behaviour [32]. Fourth, the methodological quality of a portion of studies conducted to support the IGD diagnosis has been challenged, as being based mostly on community and not clinical samples [30]. Several counterarguments have been provided. Specifically, it has been supported that IGD behaviours should be addressed dimensionally (i.e. from minimum to maximum), with symptoms following a rather normal distribution among the gamers population [10]. Thus, only a small proportion of gamers presents with diagnosable behaviours (and the medium itself is not pathologized [38];). In addition, it was argued that addictions in general (substance abuse and/or gambling) tend to constitute secondary symptoms (i.e. maladaptive behaviours addressing the distress related to pre-existing psychopathologies [39];). Therefore, and given that other addictions are classified as independent conditions, one could assume that this should be also followed in the case of IGD. Last, person focused conceptualizations have been introduced to understand ones IGD symptoms. These emphasize the significance of user related characteristics and avoid incorrect generalizations to all gamers population [39]. In the light of this unfolding dialogue among scholars in the field, the APA [22] invited further studies in relation to IGD presentations, and in particular its risk and protective factors.

### Conceptual model for understanding IGD Behaviours

This research responds to this call via adopting a holistic conceptual model to understand and study IGD behaviours followed in past research [10, 21, 40]. This suggests that IGD behaviours present a normal variability among gamers, between minimum and maximum intensity. In that line, it is assumed that ones IGD behaviours exhibited may also vary over the life-course. The latter depends on the interaction between risk and protective effects related to characteristics of the gamer, the game-application, as well as the gamers surrounding outside the game [10, 21, 40]. Adopting this notion, the present research investigates a gamers individualism-collectivism values/profile both as a likely risk and protective factor, via the implementation of an advanced profiling analysis [3]. Furthermore, it does that in relation to both ones IGD behaviours overall, as well as considering separately the nine criteria suggested by DSM-5 as defining the syndrome.

### Individualism-collectivism values and IGD

Numerous IGDassociated factors have received attention in the past [18]. These include indicativelylower levels of exercise [41], reduced self-satisfaction outside the game [8], being a male [9], and being an adolescent [42, 43]. Considering the latter in particular, a number of studies appear to imply that younger people, adolescents and emergent adults, are more at risk of IGD [10, 21, 40]. Within that context, ones individualism-collectivism values/orientation has been recognised as a parameter that may play a significant role in IGD [11, 38]. Such values areoften described as involving patterns of behaviour that are both explicitly and implicitly acquired, and are transmitted through symbols or practices, which are shared by those who accompany a common collective/social identity [44, 45]. Two main dimensions have been adopted to help explain and encompass such differences within a population, individualism, and collectivism [44, 45]. Individualism is explained asthe influence of values, where ones social context/group is viewedand experiencedrather separately from their self [44,47]. Therefore, ones own thoughts, feelings and interests play the most significant role in defining their goal-directed behaviours and decisions; and not those of the group they are members of (e.g. family, religious community) [44,47]. Examples that would identify as more individualistic societies would be nations such as Australia or the United States of America (USA [4, 47];). On the other hand, collectivist values can be described as promoting an interrelated sense of self with ones social surrounding, that is often inescapable [44,47]. Thus, one makes decisions motivated more by what is deemed as expected or beneficial for the groups they belong into (e.g. one does what their family expects from them [46, 47];). Subsequently, this type of valuesmay build social relationships less on individual attributes or self-worth but rather tends to privilege family and/or memberships of certain societal structures [44,47]. An example of a country/society that would identify as a collectivistic country would be China [46, 47]. While it has been noted that ones individualism-collectivism orientation is associated to their culture, this is not exclusive [48, 49]. For example, sociodemographic domains, such as the values and influences present in ones home and school; as well as biological and genderrelated influences, also interfere (e.g. females tend to be more collectivistic than males) [12, 48, 49].

Two additional distinctions of individualism and collectivism involve a horizontal category and a vertical category for each [46, 47]. Firstly, horizontality can be explained as assuming equality between the members of ones group, whereas verticality viewsindividuals as more or less unequal to each other [44,47]. Therefore, horizontally individualisticvalues encourage people to perceive each other as equal, whilst they are simultaneously independent of each other in terms of what drives their behaviours. Nonetheless, vertical-individualistic valuesstill assume people as independent from each other but simultaneously view them as more or less unequal (clear hierarchy [44,47];). To adapt this to collectivism, a horizontal-collectivist value system would illustrate a society where individuals are self-construal or equal but are interdependent of each other (e.g. Japan) whereas a vertical-collectivist value system demonstrates a society where people a perceived as unequal and are also interdependent of each other (e.g. India [44,47];).

Based on these, behavioural motivations have been assumed to significantly differ among people differing on individualism-collectivism [44,47]. In particular, drives of achievement are hypothesized to be more definitive for those with more individualistic values, connection and belonging for those with more collectivistic values, whereas hierarchy and competition drives as being more related to values of verticality [44, 45, 47]. Interestingly, such differences have been inferred to effect gaming engagement drives underpinning the severity of IGD behaviours [11, 20, 38]. For instance, a gamers competitive drive against others in order to climb higher within the game hierarchy (e.g. higher in-game level) has been proposed to attract gamers with more vertically individualistic values, where achievement, competition and authority ranking are established as central drivers of ones behaviour [44].

Indeed, two recent studies have confirmed such hypotheses in relation to IGD symptoms. Firstly, OFarrell and colleagues [11] examinedindividualism-collectivism orientation as a moderatorof the relationship between depression and IGD behaviours. Researchers concluded that gamers who were vertically individualistic and experienced high depression levels, in turn, experienced aggravated IGD behaviours compared to equally depressed but less vertically individualistic gamers. Second, Stavropoulos and colleagues [50] assessed a similar moderating effect of vertical individualism in the association between inattention and IGD symptoms. Their findings demonstrated an association between IGD behaviours and inattention, and additionally were exacerbated by more vertically individualistic cultural values. These findings are in line with the notion that individuals who relate to values of high independency and to an extent social disconnection, as more vertically individualistic values tend towards, are more predisposed to addictive disorders likeIGD [11, 51, 52]. It has therefore been illustrated how a vertical-individualistic orientation may increase the severity of IGD symptoms in the context of a co-existing psychopathology [11, 20]. This is implied to occur, when there is synchronization between a gamersvalue-dictated drives of hierarchy, personal rewards or competitive success and the in-game mechanics that the player experiences (e.g. levelling up and winning over another player for in-game rewards [11, 17, 51, 52];).

Despite this progress in relation to the vertical-individualism and IGD behaviours association, the state of the available empirical evidence does not yet suffice to explain why and how IGD prevalence appears to be higher among more collectivistic east Asian countries [22]. Collectivism related drives for in-game connection and team playing, likely prompted by game mechanics involving socialization and alliances, have not been directly investigated in relation to IGD behaviour, although they have been theoretically implicated [6, 12]. Additionally, the effect of ones individualism-collectivism values on their IGD behaviours has been mainly explored as an exacerbator of a pre-existing form of psychopathology (e.g. depression, anxiety, and inattention) and not as an independent direct effect [11, 20]. Lastly, the analytic approach followed by past studies emphasized only oneindividualism-collectivism dimension (e.g. vertical collectivism) and did not holistically portray gamers across all the four co-existing individualism-collectivism aspects (i.e.vertical and horizontal individualism and collectivism [11, 20, 50];). The latter is deemed to be significant as ones individualism-collectivism profile could guide more group focused and thus effective policies for IGD prevention and intervention. Such knowledge could be of particular significance among countries which are multicultural (and therefore require the implementation of cultural/value specific practices across their population) and concurrently present to be high in the consumption of internet games, such as the USA and Australia [53].

### The innovative contribution of the present research

Based on the reviewed literature, this research utilizes an online sample consisting of over 1000 gamers from the community and being assessed in relation to their individualism-collectivism orientation and IGD behaviours. These gamers derive from multi-cultural countries such as the USA and Australia to allow the findings to inspire more IGD effective and culturally responsive policies for these high in game consumption diverse populations [53]. Furthermore, the current study advances past empirical work by: a) emphasizing on the direct effect of individualism-collectivism orientation on IGD behaviours; b) assessing all four different individualism-collectivism aspects concurrently (e.g. vertical and horizontal individualism and collectivism) to profile gamers and; c) comparing the individualism-collectivism profiles of gamers revealed both in relation to ones overall IGD behaviours (i.e. assessing ones IGD symptoms dimensionally, from minimum to maximum, as they are normally distributed to the general population), as well as the nine distinct IGD criteria separately [22]. Therefore, the following questions have been introduced:

1. Are there different typologies of internet gamers based on their characteristics, as described by the Individualism-Collectivism model?
2. If yes, how do these different individualism-collectivism typologies of gamers associate with internet gaming disorder risk?

The present study will attempt to address these innovative aimsvia the employment of an advanced and accurate statistical analysis that enables the identification of homogenous subgroups within a population [54]. By undertaking this method of analysis, the study will aim to define, the number, size, features and differences between the individualism-collectivism profiles extracted, as well as their links with IGD behaviours.

## Methodology

### Participants

The cross-sectional dataset analysed entailed numerical data from 1032 individual gamers in the community (see Table1). They were assessed for their IGD behaviours online, between December 2018 and December 2019 (see procedure section), were at least 18years of age or older and held a residency in either Australia (*n*=738), the United States of America (*n*=222) or Other Globally (*n*=72). The latteralso encapsulated gamers from the United Kingdom (*n*=7), New Zealand (*n*=14). Specifically, participants ranged between 18 to 72years old with a mean age of 24years (SD=7) and a gender composition of 503 males (48.7%) and 529 females (51.3%). At the 95% confidence interval, the estimated maximum sampling error in the present study with a sample of 1032 participants were 3.11% (Z=1.96). Therefore, itsatisfied the acceptance level recommended by Hill ([55];being in the range of 4%). A power estimation was also performed using the G-Power software [Model: a-priori, linear multiple regression, R^2^ deviation from 0, effect size f2=0.15, error probability of =.05, power (1- error probability)=0.95, a non-centrality parameter =19.35, a critical F of 2.45 and an actual power of 0.951] which indicated a minimum required sample of 129 participants for the analyses aimed [56].

**Table 1:** Age, Sex and Country of Origin

Demographics		Australia (*n*=738)	Unites States (*n*=222)	Other (*n*=72)
Age		26 (*M)*	25 (*M)*	27 (*M*)
Sex	Male	503 (48.7%)	102 (45.9%)	27 (37.5%)
	Female	529 (51.3%)	120 (54.1%)	45 (62.5%)

Note 1:

Other Multicultural Countries (*n*=72; 13.3%) involved United Kingdom (*n*=7; .7%), New Zealand (*n*=14; 1.4%) and other countries (*n*=51; 4.9%)

### Instruments, materials and measures

#### Internet gaming disorder scale 9 items short form (IGDS9-SF)

In order to measure IGD symptoms, based on the respective DSM-5 diagnostic criteria, the researchers selected the Internet Gaming Disorder Scale Short Form (IGDS9-SF). This constitutes a continuous scale of measurement that reflects the severity of IGD symptoms based on a sum of scores from nine items [26]. Participants respond to these items utilising a five-point Likert scale, which varies from 1 (Never) to 5 (Very often), thus reflecting the severity of the exhibited behaviours (e.g. Do you feel more irritability, anxiety or even sadness when you try to either reduce or stop your gaming activity? [26];). The total sum isestimated by totalling all the item scores and ranging between nine to forty-five [26]. An internal reliability analysis was conducted and resulted in a Cronbachs of 0.87 and a McDonalds of .88 [57], thus inferring high consistency on the IGDS9-SF. Conditional reliability analyses related to items deletion also concluded retainment of all scale items, as reliability would decrease upon removal of any items^1^ [3, 26]. Moreover, this research utilised the IGDS9-SF because this scale was found to be suitable, valid and reliable in measuring the DSM-5s diagnostic criteria for IGD symptoms, especially across cultural contexts or variables [26, 58].

#### Individualism and collectivism scale (ICS)

Individualism-collectivism values wereassessed with the Individualism and Collectivism Scale (ICS). This was applied in order to measure four distinctions of Individualism and Collectivism [44, 45]. Specifically, the ICS consists of 16 items, with four items measuring each different dimension including: vertical individualism (VI;e.g. Winning is everything-no matter what the group I belong to thinks), horizontal individualism (HI; e.g. My personal identity, independent of others, is very important to me-but I do recognize that we are all equal), vertical collectivism (VC;e.g. It is important to me that I respect the decisions made by my groups-and I do acknowledge that their a group hierarchy that I obey to) and horizontal collectivism (HC;e.g. If a co-worker gets a prize, I would feel proud-we are together and equal [44, 45];). Each of the 16 items is answered on a nine-point scale varying from 1 (Never) to 9 (Always). The four different dimensionsdescribed are scored by summing their respective item scores (differing between four to thirty-six; four items per subscale/subdimension). Reliability analyses were also conducted for the ICS overall and resulted in a Cronbachs of 0.70 and a McDonalds of .72 [3].

### Analytical procedure

#### Number of profiles

The first research goal aimed to identify the specific number of individualism-collectivism gamer profiles as determined by the ICS within thisgamers population. Itis addressed using LCA modelling via the TIDYLPA CRAN package in the R Studio software [54]. LCA employs targeted measurements in order to identify specific homogenous subgroups within a sample. These measurements for the present study were the four distinct dimensions of the ICS scale, VI, HI, VC and HC [44, 45].

In particular, the chosen analysiscomparatively exploresa varying number of parameters that build different forms of LCA models. In this way, the potential number of differentgamer profiles can be accurately identified and described. Specifically, the differences between the gamer profiles can be identified and explained based on the means of the various indicators (e.g. average level of VI, HI, VC and HC across profiles), their variances (e.g. variability of VI, HI, VC and HCin a profile), and their covariances (e.g. co-variability of VI, HI, VC and HC across profiles). Moreover, these parameters can be assessed simultaneously andcompared as being: a) equal; b) varying or; c) zero across the different profiles in the various parameter combinations (See Table2). Concurrently, the proportion-size of each gamer profile supported is provided. Regarding the nature of differences across the profiles proposed, their description (as previously explained) is extracted via calculating and comparatively assessing the means and variances of each dimension of individualism-collectivism employed as a profiling indicator here (i.e. VI, HI, VC and HC).

**Table 2:** TIDYLPA Models

Model Number	Variances	Covariance	Interpretation
Class-invariant parameterization (CIP)	Equal	Zero	The HI, VI, HC and HI profile indicators variances occur in the same manner across the cultural types of gamers revealed, whilst they dont covary between the profiles.
Class-varying diagonal parameterization (CVDP)	Varying	Zero	The HI, VI, HC and HI profile indicators variances occur differently across the cultural types of gamers revealed, whilst they dont covary between the profiles.
Class-invariant unrestricted parameterization (CIUP)	Equal	Equal	The HI, VI, HC and HI profile indicators variances occur in the same manner across the cultural types of gamers revealed, whilst they also covary similarly between the profiles.
Class-varying unrestricted parameterization (CVUP)	Varying	Varying	The HI, VI, HC and HI profile indicators variances occur differently across the cultural types of gamers revealed, whilst they also covary differently between the profiles.

LCA actssimilarly to CFA (confirmatory analysis), hencethe fit of the different models calculated will be evaluated based on several fit indicesin order to conclude the one model with the optimum fit for the population. It has been recommended by previous research to utilise a combined use of the Akaikes Information Criterion (AIC; lower AIC indicates a better fit), the Approximate Weight of Evidence (AWE; lower AWE indicates a better fit), the Bayesian Information Criterion (BIC; lower BIC indicates a better fit), the Classification Likelihood Criterion (CLC; lower CLC may justify a better fit), the Kullback Information Criterion (KIC; lower KIC may propose a better fit) and Entropy (values above .64 are deemed acceptable [59];).

The second research goal aimed to assess the differences between the distinct profiles supported and their IGD behaviours overall, as well as per each of the nine different IGD criteria (i.e. withdrawal, preoccupation, tolerance etc). The calculation plan involved a profile mean-differences comparison approach. This, in the case of two-classes profiles entails independent sample t-test comparisons of their overall IGD scores, as well as per independent IGD criterion. If the number of profiles revealed exceeds 3, their IGD score overall differences, as well as IGD criterion specific differences involves analysis of variance (ANOVA) models, where the different profiles will be inserted as a differentiating factor.

### Procedure

This studys ethics approval was obtained from the Victoria University, Australia Human Research Ethics Committee (HRE20-079). Given that the aim of the study relates to Internet gaming populations, participants of this archival dataset were recruited from gaming related social media forums (e.g., Gamers Forum on Facebook), and online gaming communities (e.g., www.ausgamers.com; www.forums.pcgamer.com). Data collection addressed information related to ones demographics, IGD behaviours, selected psychopathological symptoms and their individualism-collectivism tendencies. Only responses of relevance to the current study aims were used. Randomization of the sequence of the measurement instruments was employed to avoid higher concentration of missing responses on certain questions positioned at the end of the survey. Leaving questions unanswered was also not permitted to prevent missing values and participants were prompted to complete all responses. Eligible participants were required to be age 18years or older and engage in internet gaming. No direct interpersonal contact was involved. Participants were able to withdraw at any time, if they felt uncomfortable or chose not to continue with the procedure without any penalty. Finally, all participants were informed that all data/responses provided would be anonymous. Informed consent was provided prior to the initiation of the survey via ticking a box.

## Results

### Latent class analysis: number of profiles and parameterization

Upon first inspection of the latent class analysis output through the R studio software, the fit indices of 24 different models, 16 classes across the CIP, the CVDP, the CIUP and the CVUP parameterizations, were calculated, evaluated and compared (see Table 2). Among these models, the first examined was decided on the basis of the lowest AIC, BIC, AWE, CLC and KIC [59]. Second, the entropy for that model was calculated. If that was below .64, the next best model according to AIC, BIC, AWE, CLC and KIC was examined [59]. Following this process, as the best fitting model was deemed the one (out of the 24 different models examined), that had concurrently the lowest AIC, BIC, AWE, CLC and KIC, whilst presenting with an entropy of above .64. Therefore, and although initially the CVUP model with 2 classes was proposed as the one with optimum fit on the basis of the Akogul & Erisoglu [59] recommendations (see data Table3); it was rejected due to an entropy of .59.

**Table 3:** LCA Models Fit

Model	Number of Classes	AIC	AWE	BIC	CLC	KIC
CIP	1	11.628.10	11845.13	11767.61	11714.10	11739.10
CIP	2	11548.09	11739.97	11612.30	11523.64	11564.09
CIP	3	11517.97	11784.48	11606.87	11483.27	11538.97
CIP	4	11491.15	11832.22	11604.76	11446.29	11517.15
CIP	5	11398.20	11813.45	11536.50	11343.55	11429.20
CIP	6	11363.16	11852.83	11526.16	11298.49	11399.16
CVDP	1	11728.10	11845.13	11767.61	11714.10	11739.10
CVDP	2	11508.47	11760.33	11592.44	11475.56	11528.47
CVDP	3	11420.94	11806.57	11549.36	11370.15	11449.94
CVDP	4	11344.73	11864.39	11517.60	11275.81	11382.73
CVDP	5	11319.52	11973.11	11536.85	11232.60	11366.52
CVDP	6	11265.20	12052.57	11526.98	11160.38	11321.20
CIUP	1	11477.56	11683.85	11546.70	11451.56	11494.56
CIUP	2	11385.82	11666.89	11479.67	11349.44	11407.82
CIUP	3	11396.12	11751.93	11514.67	11349.40	11423.12
CIUP	4	11406.80	11837.39	11550.04	11349.69	11438.80
CIUP	5	11336.25	11840.67	11504.19	11269.71	11373.25
CIUP	6	11325.67	11904.38	11518.30	11249.22	11367.67
CVUP	1	11477.56	11683.85	11546.70	11451.56	11494.56
CVUP	2	11353.47	11784.10	11496.71	11296.31	11385.47
CVUP	3	11313.47	11967.11	11530.80	11226.49	11360.47
CVUP	4	11275.84	12152.57	11567.25	11158.94	11337.84
CVUP	5	11285.38	12385.33	11650.89	11138.45	111362.38
CVUP	6	11280.11	12603.12	11719.70	11103.28	11372.11

Based on the AIC, BIC, AWE, CLC and KIC indicators, the CIUP model with two classes was then examined (see Table 3). Givens that this model had an entropy of .81, it was deemed as the best fitting model. This entropy rate is above the recommended cut off value of .64, levels below which have been linked with less than 80% probability of accurate classification [60]. Such a rate of entropy suggests that the VI, HI, VC and HC values indicators selected to inform profiles of gamers in the present study, discriminate well between the two profiles suggested and provide of over 80% membership accuracy. Furthermore, the CIUP profile with two classes supported by the present analysis indicates that the HI, VI, HC and HI profile indicators variances occur in the same manner across the two types of gamers revealed, whilst they also covary similarly between these profiles.

It is noted that the CIUP parameterization with two classes, that was appreciated as the structure with the best fit had an AWE value of 11666.89, a BIC value of 11479.67 and a sample size adjusted BIC (SA-BIC) of 11419. Furthermore, the measurement of the CVDP two-class model resulted in a X^2^ (H_0_ Loglikelihood) value equal to 5674 and *p* value of .01. A detailed outlook of the AIC, AWE, BIC, CLC and KIC fit indices for each initially tested model is provided in Table 3.

### Latent class analysis: proportions of each profile

The size of each class within the selected CVDP two profile model revealed that approximately 11% of participants fell within class one (*n=*115) and that 89% of participants were classified to class two (*n*=917).

### Portraying the individualism-collectivism profiles of gamers

The standardized means of the four types (i.e. VI, HI, VC, HC) of the indicators employed here, suggested that the two classes/profiles revealed did not significantly differ considering their vertically and horizontally individualistic tendencies (mean difference across the two classes < .02 SD for VI and HI). Nevertheless, they differed significantly across their HC (mean difference across the two classes <2 SD) and VC orientation (mean difference across the two classes approximating 1 SD). In both cases, class 1 was significantly less collectivistic than class 2. In brief, class 1 displayed an average VI score slightly above the mean (although with variability; *M=*0.18, SD=1.25) and an average HI score slightly below the mean (also with variability *M*=0.15, SD=1.30*).* However, class 1, averaged a bit more than half SD below the mean for VC (also with variability; *M=*0.59*,* SD=1.12) and around 2 SDs below the mean for HC (with lower variability; *M=*1.99, SD=0.62*).* Given that the distinctive characteristic of this profile was their low HC and VC tendencies, they were named as the Collectivism Aversive (CA) gamers profile.

**Fig. 1:**
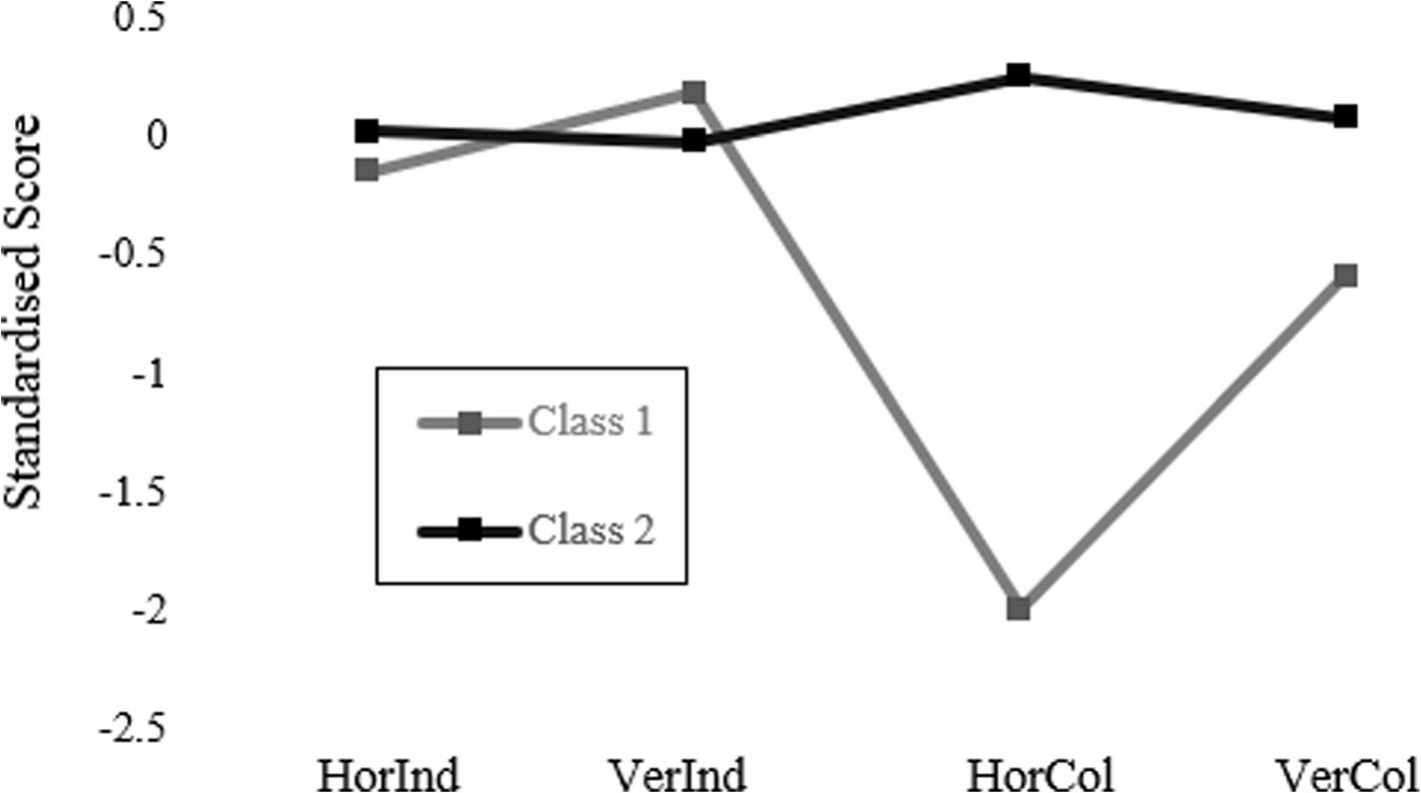
The two classes across the indicators

The results for class two have suggested that all cultural dimension scores (i.e. VI, HI, VC, HC) rather homogeneously varied within the range of one standard deviation above or below the mean. Specifically, their HC standardized average was reflected by an *M=*0.25 (SD=0.72*),* their VC average had an *M=*0.07(SD=0.96), their VC *M*=0.02 (SD=0.96) and their HI *M=*0.02 (SD=0.96). Therefore, one could say that class two displayed rather similar tendencies of all four types of cultural orientations, with a slight edge to HC. Based on their distinctive difference with class one regarding HC (and less VC), they were named as the Collectivism Neutral (CN) gamers. Table4 provides the group statistics (i.e. means and SDs) across VI, HI, VC, HC for the two profiles identified. Figure1 shows the profiles standardised means across the four individualism-collectivism dimensions.

**Table 4:** Profiles/ Classes across the four individualism-collectivism dimensions

	Cultural Profiles	N	*M*	SD	Std. Error *M*
Horizontal Individualism	Collectivism Aversive	115	0.1473	1.29947	.12118
	Collectivism Neutral	917	0.0184	.95504	.03154
Vertical Individualism	Collectivism Aversive	115	0.1773	1.25114	.11667
	Collectivism Neutral	917	0.0224	.96262	.03179
Horizontal Collectivism	Collectivism Aversive	115	1.9934	.61975	.05779
	Collectivism Neutral	917	0.2504	.71931	.02375
Vertical Collectivism	Collectivism Aversive	115	0.5930	1.12199	.10463
	Collectivism Neutral	917	0.0744	.95877	.03166

### Independent samples T-test(s): comparing the IGDS9-SF total score and separate items across the two profile of gamers

A sequence of independent samplest-tests were implemented to appreciate the differences between the CA and the CN gamers in terms of their standardized IGD total scores and their standardized 9 DSM-5 IGD criteria, as assessed via the IGD9-SF items to address the second study aim. It is noted that the 9 IGD9-SF items reflected preoccupation (item 1), withdrawal symptoms (item 2), tolerance (item 3), relapse (item 4), loss of interest (item 5), conflicts with others (item 6), deception (item 7), escapism/mood modification (item 8) and functional impairment (item 9) in relation to disordered gaming. Table5 presents the Levenes test of equality of variances results suggesting non-equal variances (Levenes test *p*<.05) between the two groups regarding both their IGD total scores, as well as all the 9 independent IGD criteria [61]. Therefore, all the t tests calculated were based on non-assumed equal variances via the interpretation of the Welchs t test [62], which is statistically adapted for this case (compared to Students t test [63];). These were computed via the Jamovi software [63, 64].

**Table 5:** Levenes Equality of Variance between CA and CN gamers in regards to IGD behaviours

	F	df1	df2	*p*
Z-IGD-Total	19.99	1	1030	<.001
Z-IGD_Q1: Preoccupation	8.64	1	1030	0.003
Z-IGD_Q2: Withdrawal Symptoms	5.69	1	1030	0.017
Z-IGD_Q3: Tolerance	14.43	1	1030	<.001
Z-IGD_Q4: Relapse	24.53	1	1030	<.001
Z-IGD_Q5: Loss of Interest	12.91	1	1030	<.001
Z-IGD_Q6: Conflicts with Others	12.53	1	1030	<.001
Z-IGD_Q7: Deception	14.72	1	1030	<.001
Z-IGD_Q8: Escapism/ Mood sModification	2.37	1	1030	0.124
Z-IGD_Q9: Functional Impairment	20.26	1	1030	<.001

A low *p*-value suggests a violation of the assumption of equal variances

**Fig. 2:**
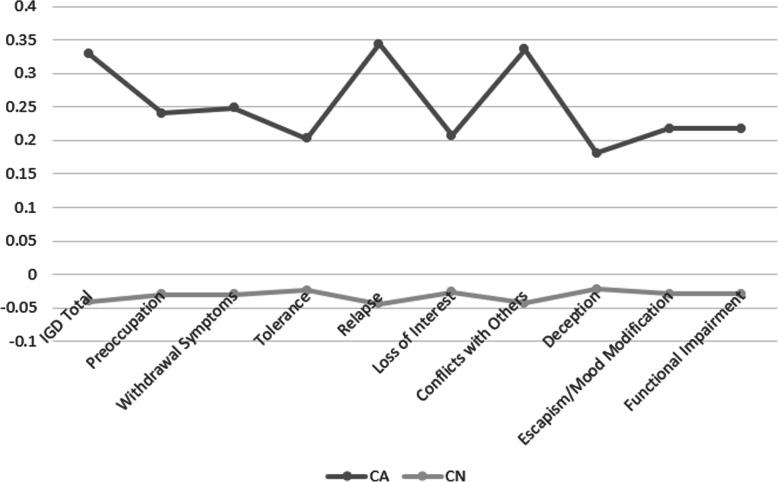
Collectivism neutral and collectivism aversive profiles across the 9 IGD criteria

Table6 presents the t-test results (Students t-test, Welchs t-test, the CA-CN mean differences and their related the effects sizes (Cohens d; Low/Small <.20; Medium<.50; Large>.80). It is noted that although the effect sizes were identical between the two criteria, given the parametric and non-equal variances sample, only the Welchs t-test was employed for the interpretation of the CA-CN mean differences. Overall, and although the CAs consistently reported higher scores compared to the CNs regarding their total IGD behaviours, as well as all the 9 separate IGD criteria; these differences were not significant considering deception (item 7) and escapism/mood modification (item 8; see Table 6). Nevertheless, the CAs were significantly higher than the CNs resulting to low moderate effect sizes between .235 (item 5; loss of interest) and .383 (item 6; conflicts with others; see Cohens d rates in Table 6) considering their IGD total score, preoccupation (item 1), withdrawal symptoms (item 2), tolerance (item 3), relapse (item 4), loss of interest (item 5), conflicts with others (item 6), and functional impairment (IGD item 9). The profile differences across IGD behaviours and the 9 IGD criteria are visualized in Fig.2. It is noted that whilst CAs consistently performed with the range between the mean and .4 SDs above the mean across both IGD behaviours overall and the 9 separate IGD criteria, the CNs ranged steadily (across both IGD total behaviours and the 9 separate IGD criteria) around .5 SDs below the mean.

**Table 6:** CA and CN gamers differences in terms of their IGD behaviors

		Statistic	df	*p*	Mean difference	SE difference	Cohens d
Z-IGDTotal	Students t	3.76^a^	1030	<.001	0.369	0.0982	0.372
	Welchs t	3.04	131	0.003	0.369	0.122	0.372
Z-IGD_Q1: Preoccupation	Students t	2.74^a^	1030	0.006	0.271	0.0988	0.272
	Welchs t	2.55	139	0.012	0.271	0.106	0.272
Z-IGD_Q2: Withdrawal Symptoms	Students t	2.82^a^	1030	0.005	0.279	0.0988	0.279
	Welchs t	2.62	139	0.010	0.279	0.106	0.279
Z-IGD_Q3: Tolerance	Students t	2.30^a^	1030	0.021	0.227	0.0986	0.228
	Welchs t	1.99	134	0.049	0.227	0.114	0.228
Z-IGD_Q4: Relapse	Students t	3.95^a^	1030	<.001	0.388	0.0983	0.390
	Welchs t	3.31	133	0.001	0.388	0.117	0.390
Z-IGD_Q5: Loss of Interest	Students t	2.37^a^	1030	0.018	0.234	0.0985	0.235
	Welchs t	1.99	133	0.049	0.234	0.117	0.235
Z-IGD_Q6: Conflicts with others	Students t	3.87^a^	1030	<.001	0.380	0.0981	0.383
	Welchs t	3.43	136	<.001	0.380	0.111	0.383
ZIGD_Q7: Deception	Students t	2.06^a^	1030	0.040	0.204	0.0989	0.204
	Welchs t	1.71	132	0.090	0.204	0.119	0.204
ZIGD_Q8: Escapism/Mood Modification	Students t	1.24	1030	0.215	0.123	0.0989	0.123
	Welchs t	1.17	139	0.246	0.123	0.105	0.123
ZIGD_Q9: Functional Impairment	Students t	2.50^a^	1030	0.013	0.247	0.0988	0.247
	Welchs t	1.99	130	0.049	0.247	0.124	0.247

^a^ Levenes test is significant (*p*<.05), suggesting a violation of the assumption of equal variances

## Discussion

In the present study, two overarching questions were proposed. These were investigated by the researchers upon inspection of the latent class analysis and the independent samples t-test that were carried out. The first question targeted whether differing classifications of internet gamers based on their individualism-collectivism characteristics could be discovered. The second question involved understanding whether these differing individualism-collectivism values classifications of internet gamers could potentially associate with IGD risk and unique symptomology. The innovative contribution of the current work in the existing body of knowledge relies on: a) the exploration of a large cohort of gamers from the community of multicultural countries, and primarily the USA and Australia, which present to be high in internet games consumption (94.6% of the sample is sourced from this two communities); b) the concurrent assessment of all four different dimensions of the individualism and collectivism model, namely vertical and horizontal individualism and collectivism [45]; c) the implementation of an advanced analytical process, which calculated and compared 24 different profiling models (i.e. equal, zero and varying variances and covariances of the indicators selected, across a number of 1 to 6 classes) to determine the one with the optimum fit and; d) the direct exploration of individualism-collectivism effects on both IGD behaviours overall as well as the nine separate IGD criteria [22].

Results revealed two distinct individualism-collectivism profiles of internet gamers. The Collectivism Aversive gamers (CAs; 11%) and the Collectivism Neutral gamers (CNs; 89%). Considering differences of the two profiles in relation to their reported IGD symptoms, the CA gamers displayed significantly higher IGD behaviours overall. Furthermore, CAs significantly outweighed CNs in preoccupation, withdrawal symptoms, tolerance, relapse, deception, escapism/mood modification and functional impairment. In contrast, the CN gamers, who displayed a more even spread of different cultural values with a slight spike of more horizontally collectivistic (i.e. group and equality orientated) behaviours presented with lower IGD symptoms. These significantly differ from those of the CAs across all the nine IGD criteria, except in relation to loss of interest and conflicts with others. Thus, the present study findings may provide user-profile centred implications for more culturally responsive prevention and treatment of IGD symptoms. Furthermore, they prompt for future research conducted in the IGD area to take into consideration the effects of individualism-collectivism profile differences among gamers.

### Understanding the cultural profiles of gamers

Analyses revealed that a class invariant unrestricted parameterization (CIUP) model of two classes/profiles best described the current sample. The models classification accuracy linked with higher than 80% probability of accurate classification for the gamers assessed across the two profiles [60]. This proposes that the VI, HI, VC and HC indicators selected to inform the profiles of gamers in the present study, discriminate well. Furthermore, the two profiles varied and covaried the same way regarding their HI, VI, HC and HC descriptors (i.e. profiles were different but equally homogeneous). In brief, the first profile accounted for 11%, whilst the second for 89% of the sample. The first profile displayed an average VI score slightly above the mean, an average HI score slightly below the mean, averaged a bit more than half SD below the mean for VC and around 2 SDs below the mean for HC. Given that the distinctive characteristic of the first profile was their low HC and VC tendencies, they were named as the Collectivism Aversive (CA) gamers profile. The second profile averaged consistently in the area of one SD above the mean across VI, HI, VC and had a slight escalation on HC. Provided their rather equal spread on VC and HC in relation to their VI and HI indicators, they were named as the Collectivism Neutral Profile.

These findings suggest the existence of two unequal in size individualism-collectivism gamer profiles, which describe a minority (CAs; 11%) and a majority of gamers (CNs; 89%). Interestingly, what was found to significantly differentiate the two profiles was their reported VC and especially HC behaviours, with the CAs being significantly lower. Thissuggests that whilst game motivations related to VI, such as achievement and authority ranking maybe present in all gamers; it is the decreased need of group orientation and group dependency that differentiates the two profiles revealed. This indeed compliments past literature suggesting that VI values are rather homogeneously related to gaming motivation (after all game achievement applies as a rather common gaming drive [11, 20, 50];). It concurrently expands the available knowledge indicating that there is a minority of gamers who are less influenced by their groups in relation to their behaviour. These gamers may also not necessarily assume/value inequality across the members of the communities they belong to. Interestingly, it is the combination of these two features that appears to distinguish the two different individualism-collectivism profiles of gamers. Nevertheless, at this point, a question should be posed. Given that this questionnaire referred to the gamers real life, it is unsure to what extent the same orientation is applied for their in-game groups. It is likely that one may compensate via their in-game groups and their in-game culture, deficits that they might experience in their real life (e.g.one participates in an in-game group, where they feel they belong, their in-game group members effect their decisions and they are all equal, to counterbalance opposite experiences they may have in their real lives [3];). Given the pioneering nature of this finding further research is invited before a solid interpretation is supported.

### Collectivism aversive and collectivism neutral gamers and IGD behaviours

The comparison of the CA and the CN gamers regarding their IGD behaviours indicated a significantly higher risk for the CAs. Specifically, whilst CAs consistently performed within the range between the mean and .4 SDs above the mean across both IGD behaviours overall and the 9 separate IGD criteria; the CNs ranged steadily (across both IGD total behaviours and the 9 separate IGD criteria) around .5 SDs below the mean. Nevertheless, the differences between the two profiles were not significant considering deception (item 7) and escapism/ mood modification (item 8; see Table 6). Conclusively, and in relation to the specific IGD criteria, the CAs were significantly higher than the CNs resulting to low moderate effect sizes regarding their preoccupation (item 1), withdrawal symptoms (item 2), tolerance (item 3), relapse (item 4), loss of interest (item 5), conflicts with others (item 6), and functional impairment (IGD item 9). These agree with past literature suggesting that such differences do interfere with IGD propensity [11, 20, 50]. Furthermore, these findings highlight that whilst achievement and higher ranking may be important for all gamers considering their gaming engagement (i.e. higher VI across both profiles); it is the lack of collectivistic tendencies that associates with higher IGD risk. This means lower influence of the group that a gamer belongs to in their behaviour and decision-making processes, and in extreme cases even disconnection from others. Such findings may indeed reflect an isolating mentality (when extremely low VC and HC scores apply). This possibility is reinforced by the fact that low HC (i.e. group and equality orientation) scores among the CN gamers appear to be their distinctive characteristic. This indeed could reflect a disillusion considering both equality with others and how essential it is for a gamer to belong in a group. These findings align with a significant body of literature suggesting that higher levels of loneliness, isolation and disconnection from others strongly associate with higher IGD behaviours [3,5, 27, 39, 65].

At this point it is noted that the two profiles appear not to significantly differ considering their IGD deception (item 7) and mood-modification behaviours. This suggests that these IGD symptoms (i.e. deception and mood-modification) may be more similarly presented among individual gamers of diverse individualism-collectivism orientations/profiles. This appears plausible in the light of past evidence suggesting the universally acknowledged emotional effect of game participation [66]. This could either reduce ones negative feelings in their real lives or even provide them with a source of positive feelings due to in game success [3, 5, 22, 23]. Considering IGD deception in particular, and based on this finding, one could assume this presents to be a rather individualism-collectivism independent behaviour of IGD symptoms. This may be due to the over-pathologizing ofinternet gaming that invites gamers to hide their real gaming time independent of the values they are defined by [30,32].

### Limitations & further research

As noted, the present study elicits various strengths including its ability to highlight the severity of IGD symptoms connected to internet gamers belonging to different individualism-collectivism profiles. However, there are several potential limitations associated with this research. Firstly, the scope of countries targeted, specifically included advanced multicultural western societies. In this way, the participants within the dataset may not be vastly representative of a wider range of cultures including ethnic populations residing in non or less multicultural and/or advanced societies. Additionally, given the heterogeneity related to the CA and CN profiles revealed, and the lack of stratification prior to the collection of the participants responses, the representativeness of the samples distribution could be further limited. Nonetheless, this possibility appears restricted, based on complex sampling error calculations conducted retrospectively. Specifically, a calculation plan involving the countries (where participants came from) as strata, ones gender, as sample weighting variable and an equal probability sampling without replacement of 1 was estimated via the SPSS 21 software [67]. Based on this plan, a cross-tabulation (X^2^) test of independence, where the two individualism-collectivism based profiles revealed, informed the columns, and ones age (i. e. emergent adults [18-29years] or not) informed the rows suggested that the samples distribution was not significantly dissimilar to the expected distribution (i. e. CA or CN* Emergent Adults or Not; X^2^=.372, *p*<.01; likelihood ratio .387, *p*<.01, odds ratio=.798;CA relative risk=.819, and CN relative risk=1.021).

Aside of sample related limitations, results were based on self-report questionnaires and therefore incorrect/ inaccurate responses, attributed to ones intention and/or reduced focus may not be excluded. The way other variables such as ones demographic characteristics could predict a gamers individualism-collectivism profile membership and its association with IGD behaviours have not been investigated. Continuing, as the study did not investigate the relationship between ones preferred internet game and individualism-collectivism orientation this may have limited the generalisations and potential findings. Lastly, given that the current sample refers to participants addressed in the community it is likely that the findings may have limited application on clinical/diagnosed IGD samples. Thus, future studies are encouraged to assess different cultural populations, emphasize on clinical samples and if possible, utilize clinical/interview assessments to compensate for these weaknesses.

## Conclusions

The investigations of this study surrounding individualism-collectivism and IGD assessment, prevention and intervention have presented significant findings for the field. First, it is indicated that different individualism-collectivism profiles of gamers do occur among populations sourced from multicultural countries and that these indeed present with different IGD risk/ propensity. Considering a gamers assessment and profiling, low collectivistic tendencies (e.g. low connectedness/dependency on ones group/community), especially when concurrently present with low horizontality (e.g. a sense of inequality among members of the same group) should be utilized as flags for likely high IGD risk.

Therefore, such individuals and groups may need to be prioritized when designing IGD-prevention initiatives. In that line, considering IGD-intervention ones therapeutic acculturation (i.e. cultural values modification) may be helpful to be targeted. Specifically, cognitive restructuring, cognitive processing (i.e. thinking about the way one thinks) and psychoeducation techniques should emphasize the value of belonging in a collective and experiencing equality with others in relation to ones mental health and gaming patterns [68]. At this point it is noted that not all CN gamers do present with IGD, given that the profiles average does not significantly exceed 2SDs from the mean across any of the indicators (although very close to that level in regard to HC). Therefore, over-pathologizing based on ones CA gamer profile should be avoided. Overall, the present findings confirm the significance of the cross-cultural psychological practice competency identified by the psychology board of Australia in the context of IGD. The latter exceeds multicultural societies and becomes even more significant given the internet gaming and IGD related global and therefore cross-cultural impact [22, 23].

## Supplementary Information

**Additional file 1.**

**Additional file 2.**

## Data Availability

Data is available in the current submission.

## Abbreviations

APA: American Psychiatric Association
CA: Collectivism Aversive
CFA: Confirmatory Factor Analysis
CN: Collectivism Neutral
CIP: Class-invariant parameterization
CIUP: Class-invariant unrestricted parameterization
CVDP: Class-varying diagonal parameterization
CVUP: Class-varying unrestricted parameterization
DSM-5: Diagnostic and statistical manual of mental disorder 5th edition
HI: Horizontal Individualism
HC: Horizontal Collectivism
ICS: Individualism Collectivism Scale
IGD: Internet Gaming Disorder
IGDS9-SF: Internet Gaming Disorder Scale 9 Items Short Form
LCA: Latent Class Analysis
VI: Vertical Individualism
VC: Vertical Collectivism
WHO: World Health Organization
